# Access to Reliable Information about Long-Term Prognosis Influences Clinical Opinion on Use of Lifesaving Intervention

**DOI:** 10.1371/journal.pone.0032375

**Published:** 2012-02-23

**Authors:** Stephen Honeybul, Kwok Ho, Susan O'Hanlon

**Affiliations:** 1 Department of Neurosurgery, Sir Charles Gairdner Hospital and Royal Perth Hospital, Perth, Western Australia, Australia; 2 Department of Intensive Care Medicine, Royal Perth Hospital, Perth, Western Australia, Australia; 3 School of Population Health, University of Western Australia, Perth, Western Australia, Australia; University Medical Center Rotterdam, Netherlands

## Abstract

**Background:**

Decompressive craniectomy has been traditionally used as a lifesaving rescue treatment in severe traumatic brain injury (TBI). This study assessed whether objective information on long-term prognosis would influence healthcare workers' opinion about using decompressive craniectomy as a lifesaving procedure for patients with severe TBI.

**Method:**

A two-part structured interview was used to assess the participants' opinion to perform decompressive craniectomy for three patients who had very severe TBI. Their opinion was assessed before and after knowing the predicted and observed risks of an unfavourable long-term neurological outcome in various scenarios.

**Results:**

Five hundred healthcare workers with a wide variety of clinical backgrounds participated. The participants were significantly more likely to recommend decompressive craniectomy for their patients than for themselves (mean difference in visual analogue scale [VAS] −1.5, 95% confidence interval −1.3 to −1.6), especially when the next of kin of the patients requested intervention. Patients' preferences were more similar to patients who had advance directives. The participants' preferences to perform the procedure for themselves and their patients both significantly reduced after knowing the predicted risks of unfavourable outcomes, and the changes in attitude were consistent across different specialties, amount of experience in caring for similar patients, religious backgrounds, and positions in the specialty of the participants.

**Conclusions:**

Access to objective information on risk of an unfavourable long-term outcome influenced healthcare workers' decision to recommend decompressive craniectomy, considered as a lifesaving procedure, for patients with very severe TBI.

## Introduction

Decompressive craniectomy has been assumed to be a lifesaving rescue treatment in severe traumatic brain injury (TBI) and ischaemic stroke, when severe brain swelling is not responsive to conservative medical therapy [1]–[5]. The surgical procedure is technically straightforward and involves removal of a large segment of the skull, either unilaterally or bilaterally, in situations where there is significant brain swelling. For many years the possibility of producing an increasing number of very severely disabled survivors after this potentially lifesaving, but non-restorative surgery, has been a major source of discussion [6], [7]. Recent trials on decompressive craniectomy for patients with ischaemic stroke have, however, demonstrated that the procedure not only increases the number of survivors but also the number of patients with a favourable functional outcome [8], [9], [10]. Furthermore, many patients who had survived the disease and procedure were also satisfied with the treatment they had received, despite they remained severely disabled [11].

Currently the same cannot be said for severe TBI [12], [13]. Traditionally, decompressive craniectomy is considered as a lifesaving procedure [14], [15], used when intracranial pressure (e.g. >25–30mmHg) is refractory to second- or third-tier medical therapy. This is because the procedure itself is associated with significant complications, some of which can be life threatening in its own right [16]. A recent multicentre trial has indeed demonstrated that early decompressive craniectomy, used when the intracranial pressure was above 20 mmHg for more than 15 minutes after the first-tier medical therapy, did not improve survival and functional outcome at 6-month after surgery [17], suggesting that this procedure should be, at best, reserved as a lifesaving procedure instead of as an early intervention in an attempt to improve long-term neurological outcome. Even when the procedure is used as a lifesaving procedure in severe TBI, the clinical effectiveness of the procedure [18] and also the associated ethical and financial issues remain highly controversial and contentious [19]–[22].

In all fields of medicine a balance between risks and benefits of an intervention is paramount to good clinical decision-making. When faced with a critically injured young person who may die imminently from intracranial hypertension, the decision to offer or withhold a potentially lifesaving, but non-restorative surgery is both emotionally and intellectually challenging for most healthcare workers. By combining the prognostic variables of age, Glasgow coma scale (GCS), pupillary reaction, extracranial injuries and radiological appearances, the CRASH trial collaborators have gone some way to address this issue by developing a user friendly web-based outcome prediction model that has been internally and externally validated in both high and low income countries [23]. By applying this model to a population-based cohort of patients who have had a decompressive craniectomy in Western Australia, we have demonstrated that although the model was not perfectly calibrated, it did provide an objective index of injury severity and the likely functional outcome at 18 months after using decompressive craniectomy as a lifesaving procedure for severe TBI [14], [15].

The question remains whether access to reliable objective prognostic information on long-term function outcome would influence clinical decision-making. Our preliminary study showed that access to objective prognostic information can influence neurosurgeons and intensivists' decision-making on using decompressive craniectomy as a lifesaving procedure [24]. Whether such information will influence the decision-making process of other non-specialist healthcare workers or lay people, including patients and their relatives, remains uncertain.

We hypothesized that access to objective prognostic information on long-term functional outcome after decompressive craniectomy for severe TBI will influence healthcare workers' opinion about whether the procedure should be offered as a lifesaving procedure, but the effect of such information may differ depending on the demographic factors and religious background of the healthcare workers.

## Methods

After obtaining hospital ethics committee approval, healthcare workers in three tertiary hospitals in Western Australia were invited to participate in this observational study. This study involved answers to written questionnaires after a presentation of three case scenarios in the form of small seminars. Given the large number of participants it was decided to obtain verbal rather than written consent to participate, as approved by the ethics committee. All patient and participant data was de-identified to maintain anonymity. An attempt was made to survey opinion amongst as wide a variety of healthcare personnel as possible. Approximately one third of the participants were neurosurgeons, intensivists and neurosurgical and intensive care nurses who were very familiar with the procedure. The remaining participants were from clinical specialties or ancillary staff with only moderate or minimal experience of dealing with these patients. The reason for including participants who have relatively little experience in dealing with these patients was to use them as ‘surrogates’ for responses of lay people or patient's relatives who also have very little experience or knowledge of the procedure but have to give the consent for the procedure in real life situations.

**Figure 1:**
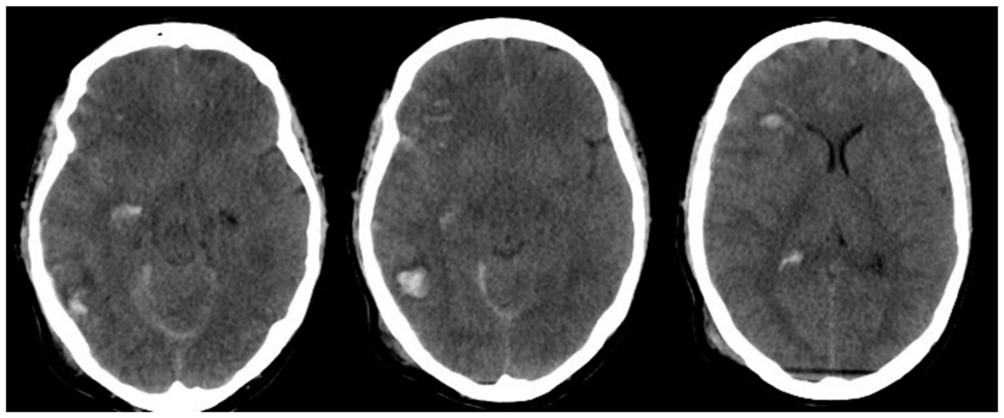
A fifty two year old female motorcyclist was involved in a motor vehicle accident. Initial Glasgow coma score was recorded as eleven (Eye: 3, Motor: 6, Verbal response: 2). Pupils were equal and reactive. She had sustained major pelvic and lower limb fractures. CT scan of the brain revealed diffuse petechial haemorrhages, traumatic subarachnoid haemorrhage, non evacuated haematoma and midline shift.

In order to maintain consistency the data was presented in the same manner on each occasion. Initially participants were shown three clinical cases and were informed that the injury severity increased with each case (**Figures 1** **,** **2** **and** **3**). The only other information regarding factors that may influence possible outcome was to highlight the initial GCS and pupillary reaction to light in each case. In all three cases the patients were intubated and ventilated in the intensive care unit and had uncontrolled and progressively worsening intracranial hypertension despite placement of a ventricular drain and maximal medical management. There was no cardiovascular instability and a repeat CT scan showed no changes. No specific information regarding outcome prediction was mentioned. Participants were not given information about the objective predicted risks of unfavourable outcomes, as estimated by the CRASH prediction model, in the first part of the structured interview.

**Figure 2:**
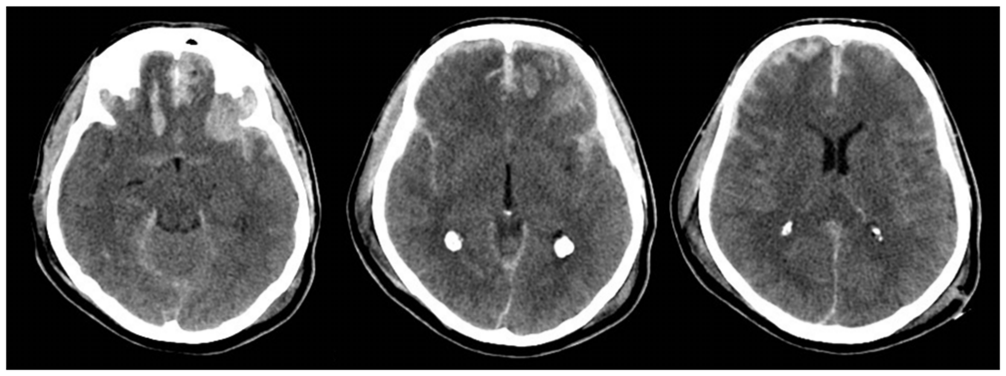
A fifty nine year old male had suffered a fall. Initial Glasgow coma score was recorded as six (Eye: 1, Motor: 4, Verbal response: 1). His right pupil was unreactive. Left pupil was reactive. There were no other injuries. CT scan of the brain revealed diffuse petechial haemorrhages, traumatic subarachnoid haemorrhage and non evacuated haematoma.

**Figure 3:**
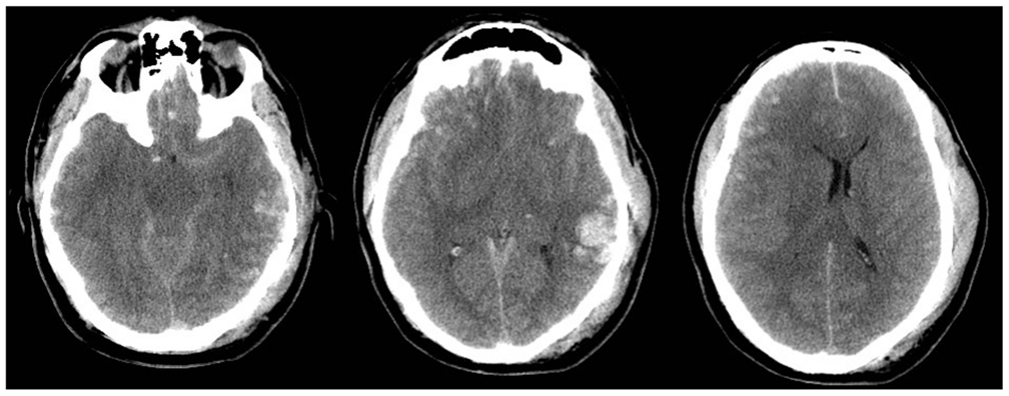
A forty two year old male with an isolated head injury following an assault. Initial Glasgow coma score was recorded as four (Eye: 1, Motor: 2, Verbal response: 1). Pupils were small and unreactive. There were no other injuries. CT scan of the brain revealed diffuse petechial haemorrhages, scattered traumatic subarachnoid haemorrhage, obliteration of the basal cistern, non evacuated haematoma and midline shift.

Using a visual analogue scale (VAS between 1 and 10) the participants were asked to write down on the chart to what degree they felt decompressive craniectomy should be performed as a lifesaving procedure based on their perception of the likely outcome. VAS of 1 signifies strongest disagreement with the decision to proceed with surgery and VAS of 10 signifies strongest agreement with the decision to proceed with surgery. The participants were then asked to what degree a decompressive craniectomy should be performed based on the following scenarios

1. The relatives or next of kin of the patients are not available.
2. The parents or next of kin of the patient are present and despite being told of the possibility of an unfavourable outcome they are insistent on any medical intervention that may improve the chance of survival.
3. The patient is a professional motorcyclist who has an advance directive that states that they would not want medical intervention that would save their life but leave them disabled.
4. The participant is in fact the patient at that age and with the presenting findings.

**Figure 4:**
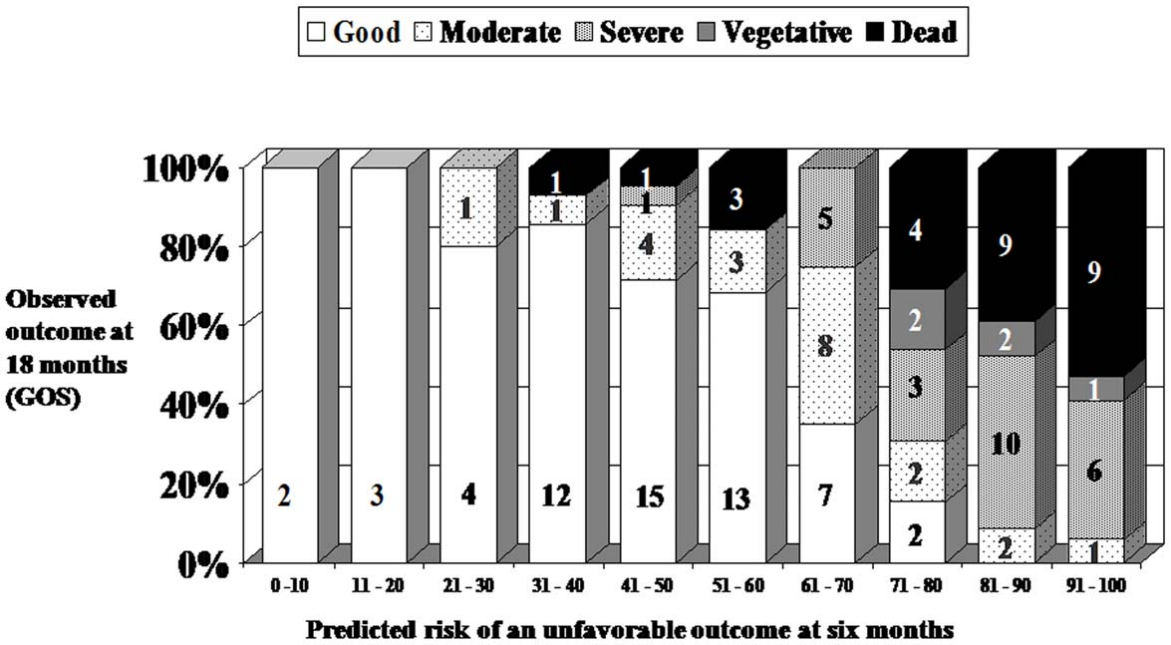
Patient outcome stratified according to injury severity. The prediction of an unfavourable outcome and the observed outcome for patients with a similar predicted risk (or index of injury severity) in a large cohort of neurotrauma patients who had had a decompressive craniectomy in Western Australia between the years 2004 and 2008 [15]. (Reproduced with kind permission, Mary Ann Liebert, Inc. publisher).

In the second part of the structured interview, the participants were shown the CRASH collaborators outcome prediction model. The parameters required for the model including country (Australia), age, presenting GCS, pupil reaction, presence of major extracranial injury, and initial CT brain findings of the three cases, together with the corresponding predicted observed risks of an unfavourable long-term functional outcome, were shown to the participants (**Table 1**)(**Figure 4**). They were then asked the same questions again and their opinions were compared with those that had been made in the first part of the interview.

**Table 1:** Patients clinical and radiological characteristics required by the CRASH prediction model, the outcome prediction and the observed outcome.

Basic model	Case 1	Case 2	Case 3
*Country*	*Australia*	*Australia*	*Australia*
*Age*	*52 years*	*59*	*42 years*
*GCS*	*11*	*6*	*4*
*Pupils react to light*	*Both*	*One*	*None*
*Major extra cranial injury*	*Yes*	*No*	*No*
**CT model**			
*Presence of petechial haemorrhages*	*Yes*	*Yes*	*Yes*
*Obliteration of third ventricle or basal*	*No*	*No*	*Yes*
*cisterns*			
*Subarachnoid bleeding*	*Yes*	*Yes*	*Yes*
*Midline shift*	*Yes*	*No*	*Yes*
*Non evacuated haematoma*	*Yes*	*Yes*	*Yes*
**Predicted outcome**			
*Risk of 14 day mortality (95% CI)*	*32.2%*	*46.8.3%*	*89.3%*
	*(20.3–46.8%)*	*(32.5–61.6%)*	*(81.5–94.1%)*
*Risk of unfavourable outcome at six months*	***72.4%***	***86.5%***	***94.4%***
	*(60.5–81.8%)*	*(78.9–91.6%)*	*(90.0–96.9%)*
**Observed outcome in patients with a**			
**similar outcome prediction (Injury**			
**severity)**			
Prediction of an Unfavourable outcome	71–80%	81–90%	91–100%
Number of patients with similar prediction	n = 13	n = 23	n = 17
Observed unfavourable outcome at 18 months	n = 9	n = 21	n = 16
*(95% CI)*	***69.2%***	***91.3%***	***94.1%***
	*(37–100)*	*(79–100%)*	*(81–100%)*

### Statistical analysis

Paired t-test was used to assess the mean differences in VAS of the participants before and after knowing the predicted risks of unfavourable outcomes of the three case patients in different scenarios. Wilcoxon rank test was used to generate the p values of the differences. Stratified analyses according to the medical specialty, age, religious background (Christianity, Hinduism, Buddhism, no religion, or others), and extent of experience in caring for similar patients in the past of the participants were also conducted.

In order to detect a difference of >1 point difference in VAS in participants' preference to proceed with decompressive craniectomy between before and after knowing the predicted and observed outcomes of the patients, a total sample size of 75 participants is required, if the standard deviation of the differences in their preference, graded by VAS, was 3. A total of 500 participants were recruited to ensure a power of >80% for the analyses of pre-defined subgroups of participants with different age and religious backgrounds. All analyses were performed by SPSS for Windows (version 13.0, 2004, IL, USA) and p value less than 0.05 was taken as significant.

## Results

A total of five hundred healthcare workers from a wide range of healthcare professions participated in this study (**Table 2**).

**Table 2:** Characteristics of the participants (n = 500).

Age groups, no (%):	
18–25	80 (16.0)
25–35	215 (42.9)
35–50	128 (25.5)
50–65	61 (12.2)
65+	6 (1.2)
Religious background, no. (%):	
None	180 (35.9)
Christian	247 (49.3)
Muslim	12 (2.4)
Buddhist	13 (2.6)
Hindu	13 (2.6)
Other	22 (4.4)
Position, no. (%):	
Neurosurgical staff	
Consultants	11 (2.2)
Registrars	9 (1.8)
Neurosurgical Nurses	67 (13.4)
Intensive care staff	
Consultants	20 (4.0)
Registrars	12 (2.4)
ICU nurses	36 (7.2)
Other Healthcare staff	
Consultants from other specialties	59 (11.8)
Registrars from other specialties	56 (11.2)
Nurses from other specialities	109 (21.8)
Allied health staff	121 (24.2)
Experience in caring or in contact with similar	
patients, no. (%):	
A lot (>20 cases)	138 (27.5)
Moderate (5–20 cases)	153 (30.5)
Minimal (<5cases)	192 (38.3)

### Opinion regarding intervention prior to knowing the objective assessment of the risks of unfavourable outcomes (Tables 3, 4, 5)

**Table 3:** Participants' opinion (mean score 1–10) regarding decompressive craniectomy for case scenario number 1.

	*Prior to seeing predicted and observed outcome*	*After seeing predicted and observed outcome*	*Difference* *	*CI*
*No family or friends*	*7.13*	*4.69*	*2.44*	*2.22–2.66*
*available*				
*Family requesting*	*7.49*	*5.44*	*2.05*	*1.84–2.26*
*intervention*				
*In the presence of an*	*4.92*	*2.97*	*1.95*	*1.73–2.17*
*advance directive*				
*If the participant*	*5.82*	*3.61*	*2.22*	*1.96–2.47*
*was the injured party*				

The prediction of unfavourable outcome: 72.4%.

*All p values <0.001 by Wilcoxon rank test.

**Table 4:** Participants' opinion (mean score 1–10) regarding decompressive craniectomy for case scenario number 2.

	*Prior to seeing predicted and observed outcome*	*After seeing predicted and observed outcome*	*Difference* *	*CI*
*No family or friends*	*5.26*	*2.72*	*2.54*	*2.35–2.74*
*available*				
*Family requesting*	*5.80*	*3.30*	*2.50*	*2.29–2.70*
*intervention*				
*In the presence of an*	*3.42*	*1.88*	*1.55*	*1.37–1.72*
*advance directive*				
*If the participant*	*3.63*	*1.95*	*1.68*	*1.46–1.89*
*was the injured party*				

Prediction of unfavourable outcome: 86.5%.

*All p values <0.001 by Wilcoxon rank test.

**Table 5:** Participants' opinion (mean score 1–10) regarding decompressive craniectomy for case scenario number 3.

	*Prior to seeing predicted and observed outcome*	*After seeing predicted and observed outcome*	*Difference* *	*CI*
*No family or friends*	*4.28*	*1.91*	*2.54*	*2.18–2.56*
*available*				
*Family requesting*	*4.80*	*2.45*	*2.50*	*2.14–2.55*
*intervention*				
*In the presence of an*	*2.70*	*1.54*	*1.55*	*1.00–1.33*
*advance directive*				
*If the participant*	*2.83*	*1.55*	*1.68*	*1.09–1.47*
*was the injured party*				

Prediction of unfavourable outcome: 94.4%.

*All p values <0.001 by Wilcoxon rank test.

There was a tendency towards advocating intervention in case one (mean score 7.1) and a tendency not to intervene in case three (mean score 4.3) reflecting the progressive increase in severity of injury from case one to case three. The family's request for intervention had little effect on the participants' opinion, although there were slightly more likely to advocate intervention. The presence of an advance directive had a more significant effect on the opinion of the participants, with more participants less likely to advocate intervention in case two (mean score 3.4) and in case three (mean score 2.7) when the severity of TBI was extremely severe.

When the participants assumed that they were the injured party, they were more likely to advocate intervention in the first case (mean score 5.82), but not in case 2 (mean score 3.42) and case 3 (mean score 2.83). Interestingly, the participants' were much less likely to advocate intervention for themselves than for their patients (mean difference in VAS −1.5, 95% confidence interval [CI] −1.3 to −1.6); their own preference was more similar to what they would do for patients with advance directives (mean difference −0.4, 95%CI −0.2 to −0.6).

### Opinion regarding intervention after knowing the objective assessment of risks of unfavourable outcomes (Tables 3, 4, 5)

Having seen the predicted and observed risks of an unfavourable outcome for each patient, there was a significant reduction in VAS of the participants' opinions in recommending decompressive craniectomy, reflecting participants' views that they would be less likely to advocate use of decompressive craniectomy as a lifesaving intervention in all three case scenarios (all p values<0.01).

The reductions in the strength of their recommendations to proceed with decompressive craniectomy after knowing the predicted and observed risks of unfavourable outcomes were consistent across different specialties, positions, prior experience in caring for similar patients, religious backgrounds and age of the participants (data available on request).

## Discussion

Our results showed that having access to objective prognostic information on long-term functional outcome of patients could influence the opinion of healthcare workers whether a potentially life saving procedure should be used. This was consistent across healthcare workers with different backgrounds and from different specialties.

The clinical decision to proceed with an intervention is usually based on a number of factors, not least of which is the possible outcome after the intervention. This study confirmed the results of our previous study that access to reliable objective prognostic information can influence healthcare workers' clinical decision-making [24]. This result has some clinical significance and requires careful consideration. First, the tendency of the participants not to recommend decompressive craniectomy after knowing the high predicted risks of unfavourable outcomes suggested that their initial perceived prognosis of the patients were better than objectively assessed by the CRASH prediction model, or alternatively the healthcare workers were unsure what the likely outcome would be and recommend a lifesaving procedure according to the ‘rule of rescue’ [20]. Previous studies have shown that healthcare workers are indeed not particularly good at understanding the prognosis of terminally ill patients and tended to offer life-sustaining treatment despite their extremely poor prognosis [25]. Whilst our findings would seem to concur with this, there are some significant differences between the two studies. The SUPPORT study involved patients who had limited life expectancy and there were numerous possible interventions over a variable time period. In addition the participants had limited data on the likely outcome of those interventions. In our study the issues to be addressed are the likely consequences of a single lifesaving intervention and the long-term quality of life after the procedure. As such, our results confirmed that long-term quality of life after a life-threatening illness is an important element in healthcare workers' decision-making about whether a lifesaving intervention is worthwhile [26]–[28] and, as such, having access to objective prognostic information may make it easier for them to make the difficult, but possibly more informed, decision for their patients.

Second, a number of studies have demonstrated that considerable conflict occurs amongst different healthcare providers in making end-of-life decisions, especially when dealing with patients with a very poor long-term prognosis [29]–[32]. Conflict between stakeholders, including doctors, nurses and relatives of the patients, often occurs because they all have different prior experience in dealing similar situation, cultural beliefs and religious backgrounds [33]. Substantial uncertainty around benefits and outcomes after a burdensome lifesaving intervention would only increase the possibility of having conflict in making what they have perceived as ‘the best decision’ for the patients [29], [33]. Our results showed that objective prognostic information had a similar effect on all healthcare workers' opinion regardless of their medical backgrounds and religious beliefs. Whilst we agree with the CRASH collaborators and others [34] that the predicted risk of an unfavourable outcome of a patient should never be used to replace clinical judgement, our results did suggest that the objective information from a reliable prognostic model may reduce conflicts among all stakeholders due to their different subjective perceptions (or hope) about the value of the lifesaving intervention.

Third, the participants' tendency to proceed with decompressive craniectomy for themselves was much lower than what they would do for their patients when there was no surrogate available is interesting. The possible reasons may include medical-legal issues, prior unpleasant experience of caring for patients with poor neurological outcomes, or a strong belief in quality of life rather than length of survival for themselves. This raises the obvious question as to whether healthcare workers are intrinsically biased in their tendency to recommend decompressive craniectomy as a lifesaving procedure for patients with severe TBI. Perhaps, the concept of ‘rule of rescue’ is so ingrained in most healthcare workers' mind that they tend to fall into the trap of dichotomizing a patient's outcome as only either life or dead [20], when in fact there is a high chance that the patient may survive in an unacceptable state to them [19], [21].

This study does have some limitations. In the first instance, whilst this study has included many non-specialist healthcare workers from different backgrounds, areas of expertises and degrees of familiarity with decompressive craniectomy, our results may not be generalizable to lay people or relatives of patients with severe TBI. Second, the participants were the sole decision-maker in the clinical scenarios in this study. In clinical practice the decision to perform a decompressive craniectomy is difficult and often involves lengthy and repeated discussions amongst a variety of different stakeholders. Whether access to objective prognostic information can reduce conflicts among stakeholders in real life clinical situations remains unproven, but this merits further investigation. Finally, this study was performed in Western Australia which is an open multicultural society. It is well established that clinical decision-making may be heavily dependent on the medical specialists' opinions in some countries and cultures [33], [35]. As such, our results may not be generalizable to countries with very different cultures and religious backgrounds from Australia.

Despite the limitations of this study, we have demonstrated that amongst a wide variety of healthcare workers there is a significant awareness that the decision to perform a lifesaving procedure for severe TBI cannot be simply dichotomized into life or death. In making the difficult decision about whether to proceed with a lifesaving, but non-restorative procedure, surrogate decision-makers for the patients will need as much objective and reliable information as possible to make a truly informed decision. Objective information about a patient's future quality of life appears to play a significant role in healthcare workers' clinical decision-making. Nevertheless, it must be emphasized that using a mathematical model based on what is now historical data will always have substantial limitations [36] and must not be used to replace judicious clinical judgement. As prehospital, surgical and intensive care therapies continue to advance, ongoing evaluation of outcomes and refinement on predictions of long-term functional outcomes is likely to become more and more important in the management of patients with severe TBI.
